# Progresses in biomarkers for cancer immunotherapy

**DOI:** 10.1002/mco2.387

**Published:** 2023-10-03

**Authors:** Xuwen Lin, Chenyu Zong, Zhihan Zhang, Weiyi Fang, Ping Xu

**Affiliations:** ^1^ Department of Pulmonary and Critical Care Medicine Peking University Shenzhen Hospital Shenzhen Guangdong Province China; ^2^ Department of Internal Medicine Shantou University Medical College Shantou Guangdong Province China; ^3^ Department of Internal Medicine Zunyi Medical University Zunyi Guizhou Province China; ^4^ Cancer Research Institute School of Basic Medical Science Southern Medical University Guangzhou Guangdong Province China; ^5^ Cancer Center Integrated Hospital of Traditional Chinese Medicine Southern Medical University Guangzhou Guangdong Province China

**Keywords:** biomarker, immunity, immunotherapy, liquid biopsy, tumor

## Abstract

Currently, checkpoint inhibitor‐based immunotherapy has emerged as prevailing treatment modality for diverse cancers. However, immunotherapy as a first‐line therapy has not consistently yielded durable responses. Moreover, the risk of immune‐related adverse events increases with combination regimens. Thus, the development of predictive biomarkers is needed to optimize individuals benefit, minimize risk of toxicities, and guide combination approaches. The greatest focus has been on tumor programmed cell death‐ligand 1 (PD‐L1), microsatellite instability (MSI), and tumor mutational burden (TMB). However, there remains a subject of debate due to thresholds variability and significant heterogeneity. Major unmet challenges in immunotherapy are the discovery and validation of predictive biomarkers. Here, we show the status of tumor PD‐L1, MSI, TMB, and emerging data on novel biomarker strategies with oncogenic signaling and epigenetic regulation. Considering the exploration of peripheral and intestinal immunity has served as noninvasive alternative in predicting immunotherapy, this review also summarizes current data in systemic immunity, encompassing solute PD‐L1 and TMB, circulating tumor DNA and infiltrating lymphocytes, routine emerging inflammatory markers and cytokines, as well as gut microbiota. This review provides up‐to‐date information on the evolving field of currently available biomarkers in predicting immunotherapy. Future exploration of novel biomarkers is warranted.

## Graphical abstract

- Although immunotherapy has dramatically changed the clinical therapeutic landscape of various cancer patients, predictive biomarkers should therefore be early identified to discriminate patients who are most likely to benefit from immunotherapy.
- This review mainly focused on recent clinical trials presenting biomarkers derived from tissue, peripheral blood as well as gut microbiome that encompass tumor cells, immune cells, solute inflammation markers, and commensal bacteria.
- More prospective clinical trials should be conducted to evaluate more noninvasive biomarkers, which might aid physicians in guiding routine immunotherapy practice.

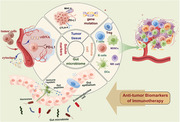

## 1 INTRODUCTION

In the management of many cancers, immunotherapy has turned out a breakthrough method to inhibit and kill tumor cells by activating immune system and increasing antitumor immunity.^1^ Immunotherapy has revolutionized traditional tumor treatment strategies, increasing the chances of survival for cancer patients. At present, immune checkpoint inhibitors (ICIs) have transformed the therapeutic landscape of immunotherapy and have been approved by the United States Food and Drug Administration ( USFDA) for a wide range of solid cancers such as melanoma, non‐small cell lung cancer (NSCLC), gastric cancer (GC), head and neck squamous cell carcinoma (HNC), and liver cancer.^1^, ^2^, ^3^ It has been shown that ICIs could disrupt immunosuppression of regulatory T (Treg) cells by targeting antibodies such as programmed cell death protein‐1 (PD‐1) and programmed cell death‐ligand 1 (PD‐L1), and cytotoxic T‐lymphocyte antigen‐4 (CTLA‐4).^4^ KEYNOTE‐001, ‐042, ‐189 have shown that immunotherapy can prolong survival in patients with advanced or metastatic NSCLC.^5^, ^6^, ^7^ As well, ICIs have shown promising results in several solid cancers.^8^, ^9^, ^10^, ^11^

The fact remains, however, that cancer patients relatively rarely benefit from ICIs, even though they have been shown to be effective in a variety of malignant tumors.^12^, ^13^ For instance, only 20−25% of patients with NSCLC respond to ICIs.^13^, ^14^ Furthermore, hyperprogression disease (HPD) patterns are increasing among patients being treated.^15^ Along with being effective against tumors, immunotherapy can also cause significant immuno‐related adverse events, resulting in increased mortality, as well as the cost of healthcare.^16^ Thus, it is unmet need to identify predictive clinical and biological biomarkers of response to ICIs in order to prescribe them more selectively and to better understand their mechanisms of action. Currently, certain established tissue‐based biomarkers, such as PD‐L1 expression and tumor mutation burden (TMB), have demonstrated a positive association with enhanced response rates in specific tumor categories. However, it is noteworthy that some patients with TMB‐low and PD‐L1 negative tumors also exhibit significant treatment responses.^17^ Moreover, the timing of the biopsy and the considerable variability in the tests have presented constraints, thereby emphasizing the necessity of devising innovative approaches that utilize more dependable indicators associated with ICIs.

Increasing evidence suggests that various factors, such as the tumor microenvironment (TME), tumor immunogenicity, antigen presentation, and oncological signaling pathways, play significant roles in both immunotherapy resistance and response mechanisms.^17^ An increasing number of biomarker‐driven therapies are becoming accessible for the treatment of metastatic disease. Here we provide a comprehensive overview of advances in utilizing biomarkers derived from tumor and systemic immunity to modulate tumor response to immunotherapy. A range of host factors, encompassing the status of tumor and solute PD‐L1, microsatellite instability (MSI), TMB, oncogenic signaling, epigenetic regulation, circulating tumor DNA (ctDNA) and infiltrating lymphocytes, routine emerging inflammatory markers and cytokines, as well as gut microbiota, have been extensively investigated.

## 2 BIOMARKERS OF IMMUNOTHERAPY IN TUMOR TISSUE

**FIGURE 1:**
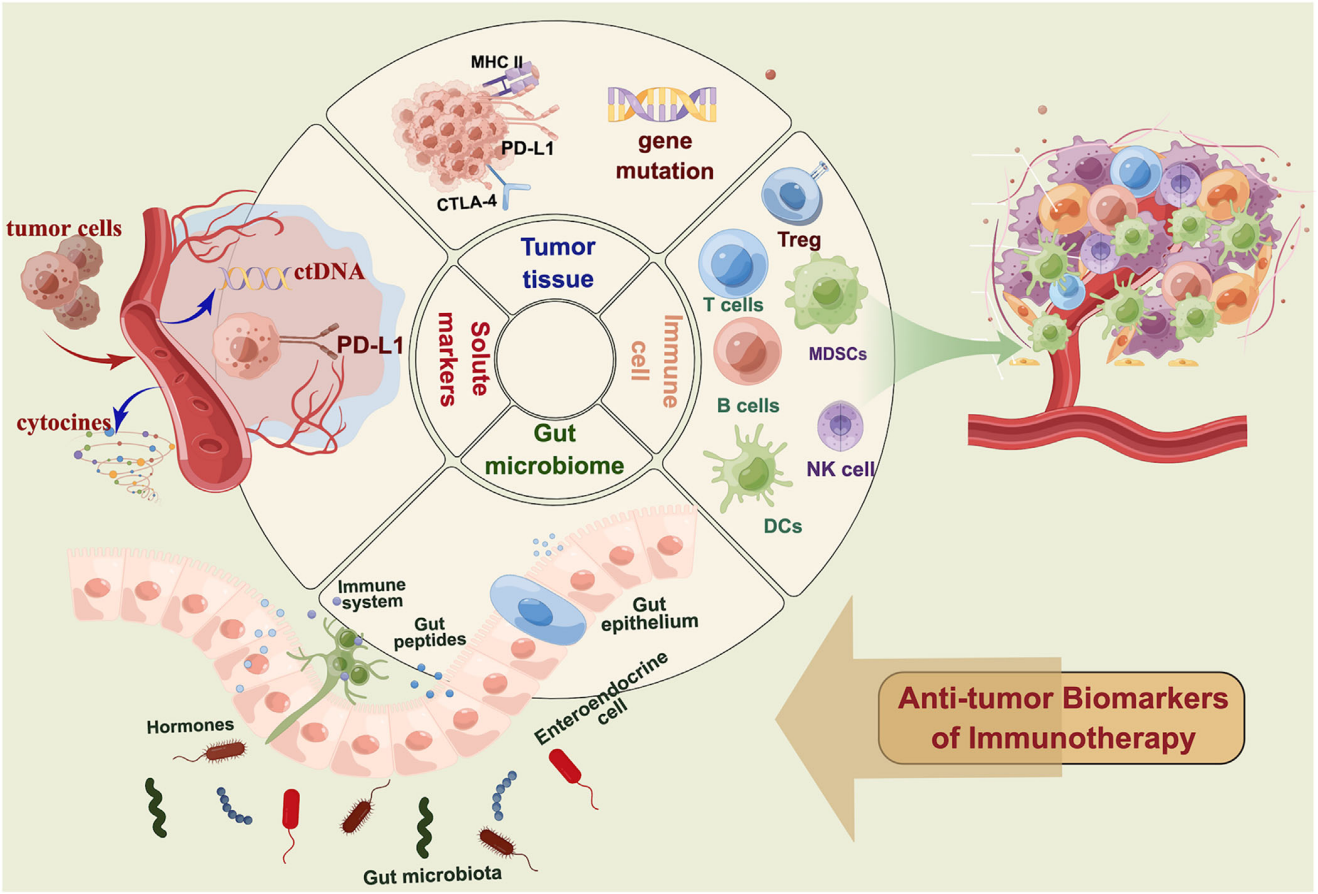
Potential predictive biomarkers of response for antitumor immunotherapy. Biomarkers could be found from tumor tissue biopsy samples, peripheral blood, and gut microbiome. Biomarkers isolated from tumor biopsy mainly include PD‐L1 expression, TMB, driver gene mutation, oncogenic signaling, and epigenetic regulation. Peripheral blood biomarkers include immune cells (such as T, B, NK, DCs, MDSCs, and Treg cells) and solute markers (such as ctDNA, soluble PD‐L1). Gut microbiome is also a promising predictor for immunotherapy. PD‐L1, programmed cell death ligand‐1; CTLA‐4, cytotoxic T‐lymphocyte antigen‐4; TMB, tumor mutation burden; HLA, human leukocyte antigen; ctDNA, circulating tumor DNA; bTMB, blood‐based tumor mutation burden; NK cells, natural killer cells; DCs, classical dendritic cells; MDSCs, myeloid‐derived suppressor cells; Treg, regulatory T; TCR, T cell receptor; MHC, major histocompatibility complex (www.home‐for‐researchers.com).

In contemporary oncology, prevalent markers utilized in the assessment of tumor tissue encompass PD‐L1 expression level, TMB, deficient mismatch repair (dMMR)/MSI, and distinct driver gene mutations. Recent progressions in comprehending the intricacies of epigenetic and oncogenic mechanisms have unveiled their pivotal role in governing the antitumor response at the interface between cancer cells and T‐cells. Herein, we undertake a comprehensive review of potential immunotherapy biomarkers derived from tumor tissue (Figure 1).

### 2.1 Immune checkpoints molecule: PD‐L1 expression

As of today, PD‐L1 expression is one of the most commonly used predictive biomarkers assessed by immunohistochemistry of tumor tissue biopsy in solid tumors.^17^, ^18^, ^19^ The overexpression of PD‐L1 has been a common strategy used by tumor cells to escape immune system.^20^ This suggests that PD‐L1 expression may play a crucial role in suppressing T‐cell immunity responses. There is a correlation between tissue PD‐L1 expression and therapeutic effectiveness in a number of initial clinical trials. Table 1 summarizes the results of each study mentioned above. In KEYNOTE‐028, 475 patients with PD‐L1‐positive advanced solid tumors showed a significant improvement in pembrolizumab objective response in patients with high levels of PD‐L1.^21^ As well, KEYNOYE‐024 and KEYNOYE‐001 showed that pembrolizumab was associated with significantly superior overall survival (OS) over chemotherapy in patients with advanced NSCLC and PD‐L1 ≥ 50%.^5^ ICIs treatment in second‐line metastatic NSCLC and melanoma clinical trials prolonged survivals over chemotherapy, though greater benefits were observed in tumors with higher PD‐L1 levels.^9^, ^22^, ^23^, ^24^ In spite of substantial evidence that indicates PD‐L1 expression correlates with therapeutic efficacy, some clinical studies report a lack of direct correlation between PD‐L1 and response.^6^, ^25^ A number of phase 3 trials (Check‐Mate‐017/‐057, KEYNOTE‐042/‐189) have shown that nivolumab or pembrolizumab monotherapy produces prolonged OS benefits in NSCLC beyond chemotherapy, irrespective of PD‐L1 expression level.^6^, ^7^, ^26^ As noted previously, chemotherapy agents have the potential to enhance tumor immunogenicity by modulating the immune system.^2^ There are currently numerous clinical trials combining immunotherapy and chemotherapy in an attempt to raise patients' response rates. In contrast to chemotherapy alone, pembrolizumab plus chemotherapy improved outcomes among PD‐L1‐negative NSCLC patients studied by Borghaei et al.^27^ Nonetheless, apart from being significantly associated with tumor response in KEYNOTE‐224, there has not been a clear association reported for hepatocellular carcinoma (HCC).^11^, ^28^ Moreover, as recently reported, nivolumab plus ipilimumab improved 5‐year OS in Check‐Mate 227 over chemotherapy, regardless of PD‐L1 expression.^29^ Data from recent large pooled analyses have reinforced the long‐term benefits inhibited previously with nivolumab plus ipilimumab regardless of PD‐L1 expression and histologies.^12^, ^29^, ^30^ Evidence reviewed here indicates contradictory and concerning results between treatment with ICIs and PD‐L1 expression.

**TABLE 1:** Main studies of tumor PD‐L1 expression to predict immunotherapy in the last 5 years.

Cancer type	Study design	Number of patients	Assay	Therapy	Key findings	References
Dependent of PD‐L1 expression						
Treated/untreated NSCLC	KEYNOYE‐001, phase 1	550	IHC, 22C3	Pembrolizumab	PD‐L1 TPS ≥ 50% was associated with longer mOS vs. TPS 1−49%	Leighl et al.^5^
Untreated NSCLC	KEYNOTE‐042, phase 3	1274	IHC, 22C3	Pembrolizumab/platinum‐based chemotherapy	OS was better for pembrolizumab than chemotherapy in all PD‐L1 TPS groups (≥50, ≥20, and ≥1%).	Mok et al.^6^
Treated melanoma	KEYNOTE‐151, phase 1b	103	IHC, 22C3	Pembrolizumab	Median OS was associated with PD‐L1‐positive (1 vs. <1%).	Si et al.^9^
20 solid tumor^a^	KEYNOTE‐028, phase Ib	475	IHC, 22C3	Pembrolizumab	PD‐L1 CPS > 1% and TMB were correlated with higher ORR and PFS.	Ott et al.^21^
Treated NSCLC	KEYNOTE‐033, phase 3	425	IHC, 22C3	Pembrolizumab/docetaxel‐based chemotherapy	Longer OS was associated with increasing PD‐L1 TPS ≥ 1%.	Ren et al.^22^
Untreated NSCLC	KEYNOTE‐024, phase 3	305	IHC, 22C3	Pembrolizumab/platinum‐based chemotherapy	PD‐L1 TPS ≥ 50% was associated with superior OS and mPFS for pembrolizumab.	Reck et al.^23^
Treated NSCLC	POPLAR, phase 2, OAK phase 3	287, 1225	IHC, 22C3	Atezolizumab/docetaxel‐based chemotherapy	Longer OS was associated with increasing PD‐L1 TPS ≥ 1%.	Mazieres et al.^24^
Independent of PD‐L1 expression						
Untreated nonsquamous NSCLC	KEYNOTE‐189, phase 3	410	IHC, 22C3	Pembrolizumab+pemetrexed/platinum‐based chemotherapy	OS, PFS, and ORR benefit was better in pembrolizumab + pemetrexed vs. chemotherapy group, regardless of PD‐L1 (TPS 1 vs. < 1%).	Rodriguez‐Abreu et al.^7^
Untreated HCC	KEYNOTE‐224, Phase 2	51	N/A	Pembrolizumab	Durable responses were observed in PD‐L1+ expression (<1 vs. ≥1%)	Verset et al.^11^
Untreated NSCLC	CheckMate 227, 817, 568, and 012	1332	IHC, 28‐8	Nivolumab + ipilimumab	Durable survival benefit with nivolumab + ipilimumab were revealed regardless of tumor PD‐L1 (<1 vs. ≥1%) and histology.	Borghaei et al.^12^
Treated NSCLC	CheckMate 017, 057, phase 3	272, 582	IHC, 28‐8	Nivolumab/platinum‐based chemotherapy	Durable responses were observed with nivolumab independent of histology and PD‐L1 TPS (≥1, ≥5, and ≥10%).	Borghaei et al.^26^
Untreated PD‐L1(‐) NSCLC	KEYNOTE‐021, 189, 407	1328	IHC, 22C3	Pembrolizumab+chemotherapy/chemotherapy	Pembrolizumab plus chemotherapy improved OS and PFS over chemotherapy (PD‐L1 TPS < 1%).	Borghaei et al.^27^
HCC	GO30140, phase 1b	223	IHC, SP263	Atezolizumab with or without bevacizumab	PFS benefit was observed in atezolizumab plus bevacizumab irrespective of PD‐L1 status (three different cutoff TPS ≥ 1, ≥5, and ≥10%).	Lee et al.^28^
Untreated NSCLC	CheckMate 227	1739	IHC, 28‐8	Nivolumab + ipilimumab/nivolumab/chemotherapy	Nivolumab plus ipilimumab increased 5‐year OS and DCB vs. chemotherapy, regardless of PD‐L1 expression (<1 vs. ≥1%).	Brahmer et al.^29^
Untreated NSCLC	CheckMate 9LA, phase 3	719	IHC, 28‐8	Nivolumab plus ipilimumab/chemotherapy	OS, PFS, and ORR was greater in nivolumab plus ipilimumab vs. chemotherapy, regardless of PD‐L1 TPS and histology (<1 vs. ≥1%).	Paz‐Ares et al.^30^

ICIs, Immune checkpoint inhibitors; PD‐1, programmed cell death protein‐1; PD‐L1, programmed cell death‐ligand 1; CTLA‐4, cytotoxic T‐lymphocyte antigen‐4; NSCLC, non‐small cell lung cancer; mOS, median overall survival; HCC, hepatocellular carcinoma; PFS, progression‐free survival; TPS, tumor cell proportion score; CPS, combined positive score; IHC, immunohistochemistry; SP263, VENTANA PD‐L1; DCB, durable clinical benefit; ORR, objective response rate.

^a^20 solid tumor: anal canal squamous cell carcinoma, biliary tract adenocarcinoma, breast cancer, carcinoid tumors, cervical squamous cell carcinoma colon/rectal adenocarcinoma, endometrial, esophageal carcinoma, glioblastoma multiforme, leiomyosarcoma, mesothelioma, nasopharyngeal, neuroendocrine, ovarian epithelial cancer, pancreatic adenocarcinoma, prostate adenocarcinoma, salivary gland, small‐cell lung carcinoma, thyroid cancer, vulvar small‐cell lung carcinoma.

The assessment of the status of PD‐L1 expression remains technical and biological limitations due to a variety of factors. They may, for example, be influenced by spatial intratumoral heterogeneity, temporal effects, differing definition, and different thresholds lack of standardization.^31^, ^32^, ^33^ This complicates the decision regarding when and where a biopsy should be performed. In this regard, it is worth noting that the overall burden of tumor may be more indicative of PD‐L1 expression in newly acquired biopsies and whole‐tissue samples in resected tumors.^31^ As a consequence, it is extremely important to learn about these limitations in PD‐L1 expression when used as potential biomarkers that might enable predicting ICI efficacy.

### 2.2 Tumor mutation burden

Since neoantigen accumulation can increase the chances of tumor cells being recognized by infiltrating immune cells, TMB could result in neoantigen production and further reinforce the antitumor immune response. Based on preclinical data, it is, therefore, more likely to respond to ICIs.^34^, ^35^ Previous studies have shown a positive correlation between TMB levels and response to ICIs in a wide variety of tumor types (Table 2). US FDA approval of pembrolizumab for previously progressed solid tumors in the tumor‐agnostic patients with high tTMB (≥10 Mut/Mb) diagnosed by FoundationOne CDx based upon KEYNOTE‐158.^36^ Notably, this multicohort study confirmed durable responses in patients (TMB ≥ 10 muts/Mb vs. <10 muts/Mb) across 10 treated malignancies (ORR of 29 vs. 6.0%), regardless of PD‐L1 expression.^36^ Likewise, TMB has also been incorporated into National Comprehensive Cancer Network (NCCN) guidelines as a promising indicator of ICIs (ipilimumab + nivolumab or nivolumab monotherapy) on the basis of CheckMate‐227^37^ and CheckMate‐568.^38^ Recent research builds on 12 clinical trials across 24 previously progressed solid tumors shown that high t‐TMB (≥175 mutations/exome) detected by whole‐exome sequencing (WES) (well aligned with ≥10 Mut/Mb assessed by FoundationOne CDx) was predictive of outcomes to pembrolizumab but not of chemotherapy.^39^ Importantly, this benefit occurred irrespective of PD‐L1 expression, MSI, or even the type of tumor. In light of above data, TMB seems to be a robust biomarker of ICI therapy and independent of other confounding variables, especially PD‐L1 expression.

**TABLE 2:** Main studies of tumor TMB to predict antitumor immunotherapy in the last 5 years.

Cancer type	Study design	Number of patients	Assay	Therapy	Key findings	References
10 solid tumors^a^	KEYNOTE‐158, phase 2	1066	FoundationOne CDx	Pembrolizumab	Higher ORR were observed in patients with a TMB ≥ 10 muts/Mb vs. <10 muts/Mb (29 vs. 6.0%)	Marabelle et al.^36^
Untreated NSCLC	CheckMate 227, phase 3	1004	FoundationOne CDx	Nivolumab plus ipilimumab/nivolumab/chemotherapy	Patients with high TMB (≥10 muts/Mb) showed longer PFS with nivolumab plus ipilimumab than with chemotherapy, irrespective of PD‐L1.	Hellmann et al.^37^
Untreated NSCLC	CheckMate‐568, phase 2		FoundationOne CDx	Nivolumab plus ipilimumab	Patients with high TMB (≥10 muts/Mb) showed higher ORR with nivolumab plus ipilimumab than with chemotherapy, irrespective of PD‐L1.	Ready et al.^38^
Pan‐tumor	KEYNOTE‐158, phase 2	2234	FoundationOne CDx	Pembrolizumab/ chemotherapy	TMB ≥ 175 mutations/exome was associated with longer ORR, PFS, and OS of pembrolizumab but not of chemotherapy, regardless of PD‐L1	Cristescu et al.^39^
Untreated NSCLC	DFCI,MSKCC,SU2C, retrospective	1552	OncoPanel (DFCI) and MSK‐IMPACT (MSKCC) NGS platforms	PD‐1/PD‐L1 blockade	High TMB (>19.0 Mut/Mb) and PD‐L1 expression (≥50%) have better ORR and OS. Increasing TMB levels are associated with CD8‐positive, PD‐L1+ T‐cell‐mediated response.	Ricciuti et al.^41^
Treated/untreated NSCLC	Pooled analysis of KEYNOTE‐021, −189, and −407	1298	FoundationOne CDx	Pembrolizumab Plus Platinum‐Based Chemotherapy	First‐line pembrolizumab plus platinum‐based chemotherapy or chemotherapy alone did not significantly affect tumor TMB in NSCLC.	Powell et al.^42^
CRC, EGC, HNSCC, Melanoma, NSCLC	DFCI, DFCI/PROFILE; TCGA, MSKCC	8193	WES	Anti‐PD‐1/PD‐L1 or anti‐CTLA‐4	After recalibrating TMB by regression‐based analysis, TMB‐c (true TMB‐high) provides a better outcome for people treated with ICIs than noncalibrated TMB.	Nassar et al.^43^

ICIs, immune checkpoint inhibitors; PD‐1, programmed cell death protein‐1; PD‐L1, programmed cell death‐ligand 1; CTLA‐4, cytotoxic T‐lymphocyte antigen‐4; TMB, tumor mutation burden; DFCI, Dana‐Farber Cancer Institute; MSKCC, Memorial Sloan Kettering Cancer Center; SU2C, Stand Up To Cancer; WES, whole‐exome sequencing; CRC, colorectal cancer; EGC, esophagogastric cancer; HNSCC, head and neck squamous cell carcinoma; NSCLC, non‐small cell lung cancer; TCGA, The Cancer Genome Atlas; OS, overall survival; PFS, progression‐free survival; DCB, durable clinical benefit; ORR, objective response rate.

^a^10 solid tumors: anal squamous cell carcinoma (cohort A); biliary adenocarcinoma (except ampulla of Vater cancers; cohort B); neuroendocrine tumors of the lung, appendix, small intestine, colon, rectum, or pancreas (cohort C); endometrial carcinoma (except sarcomas and mesenchymal tumors; cohort D); cervical squamous cell carcinoma (cohort E); vulvar squamous cell carcinoma (cohort F); small‐cell lung carcinoma (cohort G); malignant pleural mesothelioma (cohort H); papillary or follicular thyroid carcinoma (cohort I); or salivary gland carcinoma, excluding sarcomas and mesenchymal tumors (cohort J).

Furthermore, TMB alone has not been routinely used as a treatment decision, since there is no standardization among sequencing platforms or thresholds used to determine “high” TMB.^40^ As of yet, TMB thresholds have been used at varying levels in different studies. According to a study of 1552 patients with advanced NSCLC, TMB distributions were first normalized, and a regression tree was then fitted to model the response to ICI based on normalized TMB. The results showed high TMB (>19.0 Mut/Mb) and high PD‐L1 expression (≥50%) were associated with a better ORR than low TMB (≤19.0 Mut/Mb) and low PD‐L1 expression (<1%).^41^ Interestingly, through immunofluorescence, this research empirically investigated ICIs response mechanism of high TMB levels associated with CD8+ cell infiltration and PD‐L1 positive T‐cell infiltration. Moreover, these findings support the hypothesis that TMB may upregulate innate and adaptive immune responses, thus causing NSCLC to be more responsive to ICIs regardless of PD‐L1 expression. This study, however, is retrospective, therefore further prospective evaluation is required. Furthermore, while these promising results support the role of TMB as a biomarker for ICIs, long‐term benefits remain uncertain and inconsistent. As opposed to the preliminary results of the CheckMate 227 trial, nivolumab and ipilimumab did not have as great a benefit in patients with high TMB and high PD‐L1.^12^ In addition, a pooled analysis of KEYNOTE‐021, ‐189, and ‐407 found that tumor TMB did not significantly affect the efficacy first‐line pembrolizumab plus chemotherapy in NSCLC.^42^ There is still uncertainty regarding the extent to which TMB‐high will be an accurate biomarker for diverse patient populations. This issue has been addressed by TMB recalibration (TMB‐c) studied from Nassar et al.,^43^ which had a notable impact on decisions made concerning ICIs. It was found that tumor‐only assays can overestimate TMB, especially in African and Asian‐ancestry tumors, compared with gold‐standard assays that paired tumors with nontumors. Interestingly, when TMB was recalibrated by regression‐based analysis, TMB‐c (true TMB‐high) appears to have a better outcome than noncalibrated TMB in people treated with ICIs. Specifically, TMB‐c groups had significantly longer survival rates than did the other two groups (false TMB‐high and true TMB‐low groups). This suggests that TMB prediction value could be improved by assessing a tumor's mutational profile. An improved correlation between TMB and immunotherapy response exists when the estimate of TMB is corrected.

Furthermore, while the US FDA has approved two nongenome sequencing methods, namely MSK‐IMPACT and FoundationOne CDx assay, there are significant discrepancies in the gene panels employed in different research studies.^44^ The complexity and cost associated with WES have led to the widespread use of targeted next‐generation sequencing (NGS) in research on the TMB, resulting in varying cutoff values for this biomarker.^45^, ^46^ Consequently, when evaluating studies that investigate the TMB as a biomarker, it is crucial to take into account the variations in methodologies employed. Therefore, future studies should aim to establish a more accurate and widely acknowledged methodology for assessing the TMB in diverse cancer types.

### 2.3 Microsatellite instability

MSI which is caused by DNA dMMR, represents hypermutator phenotype in some solid cancers.^47^, ^48^ MSI‐High (MSI‐H)/dMMR‐positive tumors are at risk for somatic mutations in response to neoantigens and may infiltrating lymphocytes and upregulate the immune system, particularly tumor cells.^49^ In prior prospective trials, MSI‐H has been identified for the first time in metastatic colorectal cancers (MCRC) to be an efficacious biomarker of ICI efficacy across a wide range of malignancies.^50^, ^51^ On the basis of these results reported unprecedented ORRs in varying MSI‐H/dMMR tumors, the US FDA subsequently granted accelerated the first therapeutic agnostic approval in patients with MSI‐H/dMMR metastatic solid tumors, independent of the tumor histology. MSI‐H/dMMR has subsequently been positively correlated with durability of ICI efficacy in increasingly substantial clinical trials, in particular, MCRC, GC, and endometrial carcinoma.^52^, ^53^, ^54^ Most recently, NCCN guidelines have recommended ICIs as first‐line treatment for MSI‐H/dMMR MCRC patients on the basis of KEYNOTE‐177 trial demonstrated superior ORR and PFS in pembrolizumab over chemotherapy.^55^ Aside from MCRC, MSI is also found in other cancer types, including NSCLC, UCC, pancreatic adenocarcinoma, melanoma, and breast cancer, which also benefit from ICIs.^3^, ^10^, ^49^, ^56^ Based on an updated analysis of phase II, multicohort KEYNOTE‐158 study, patients with 27 types of advanced noncolorectal tumors with MSI‐H/dMMR showed impressive durable responses to pembrolizumab, supporting the predictive value of MSI‐H/dMMR (ORR: 30.8%, median OS: 47.5 m).^57^ In the phase II PHAEDRA study, durvalumab indicates an ORR of 40% in dMMR patients with endometrial cancer.^58^ Moreover, according to recent studies, MSI increases the effectiveness of immunotherapy for NSCLC.^59^ However, despite this, the frequency of MSI reported in NSCLC and other tumor types is very heterogeneous, resulting in the clinical application of MSI are still unclear and not yet used in clinical practice.^50^ Moreover, some studies reported that high rates of TMB without dMMR were observed in melanoma and NSCLC, indicating that using MSI/dMMR status alone to identify most patients who would more likely to benefit from ICIs.^8^, ^49^, ^60^, ^61^, ^62^ As of yet, there are still larger prospective studies required to confirm that MSI can be a reliable predictor of ICI benefits in various cancer patients.

Optimizing MSI testing in clinical practice is necessary for maximizing its predictive value. As of yet, no standards have been made in terms of the molecular test in MSI detection. In spite of the fact that IHC is the most commonly used method, several studies have shown discrepancies between polymerase chain reaction (PCR) and NGS results.^54^, ^63^, ^64^ Sustainable evidence supports that NGS is the relatively more sensitive option for MSI detection since it detects both MSI and a wide panel of other mutations, including TMB, simultaneously. Data from a comparative research study involving 138 MCRC patients suggest that NGS‐derived MSI assessments may provide superior results to IHC.^54^ However, in contrast to NGS, PCR and IHC are relatively more affordable and widespread, allowing diagnostic pathologists to score tumor cells directly even with low tumor purity.^65^ It is currently necessary to optimize MSI/dMMR detection in practice in order to identify tumor patients who are eligible for ICIs.

### 2.4 Oncogenic signaling pathways

Recent research has provided compelling evidence that oncogenes significantly influence the TME in the context of immunotherapy, and that they can benefit from treatment with immune ICIs.^66^, ^67^ The intrinsic signaling pathways within the TME play a crucial role in maintaining immunosuppressive properties and facilitating immune evasion by tumors. Notably, the phosphatase and tensin homolog deleted on chromosome ten (PTEN), phosphoinositide 3‐kinases (PI3K), and Wnt pathways have been identified as key contributors to immune exclusion, immune dysfunction, and the recruitment and differentiation of immune‐suppressive cells in various types of tumors. It has been reported that patients with melanoma who lack PTEN have fewer tumor‐infiltrating T cells and poor response to ICIs.^67^ However, there was no observed correlation between the presence of class I major histocompatibility complex (MHC) or PD‐L1 expression and the status of PTEN, indicating the existence of an alternative immune evasion pathway subsequent to PTEN loss. Experimental investigations in murine models have demonstrated that PI3K inhibitors enhances the efficacy of ICIs, and ongoing clinical trials are investigating this phenomenon. Additionally, it is hypothesized that Wnt signaling may contribute to the exclusion of T cells from tissues affected by HNC, GC, and related malignancies.^67^

Moreover, there is a considerable body of research currently underway to assess the influence of EGFR mutations on the effectiveness of ICIs. Notably, a large‐scale study has reported that patients with EGFR mutations exhibit poor OS when treated with ICIs, regardless of their PD‐L1 expression levels.^68^ Similarly, Negrao et al.^67^ have also observed that NSCLC patients with EGFR mutations do not derive additional benefits from ICIs. Consistent with the findings of the MMUNOTARGET registry, NSCLC patients with EGFR exon 21 mutations experience longer PFS compared with those with exon 19 mutations.^66^ As well as EGFR, patients with HER2 mutations also benefit less from ICIs.^67^ The aforementioned studies provide evidence that mutations in EGFR and HER2 have the potential to increase the immunogenicity and immune response of ICIs. In contrast, Negrao et al.^67^ found that patients with BRAF mutations exhibit higher levels of TMB and PD‐L1 expression, suggesting that they may be more responsive to ICIs. Conversely, KRAS mutations have been found to be positively associated with the therapeutic effects of ICIs in cancer patients.^69^ Contrary to this, the study conducted by Adi Kartolo et al.^70^ did not yield significant findings regarding the benefits of KRAS mutation. Additionally, it is important to highlight that the KRAS–G12C mutation has been found to inhibit OS and PFS to a greater extent, whereas the KRAS–G12V mutation is associated with a shorter OS. This observation leads us to speculate that the presence of other comutations in KRAS, such as STK11/KEAP1 and TP53, may contribute to distinct subgroups with varying biological phenotypes and responses to immunotherapy.^71^, ^72^, ^73^, ^74^

In conclusion, the strategy of targeting oncogene addiction to augment immune response is a formidable approach. Nevertheless, a comprehensive understanding of how these pathways regulate immune evasion, resulting in primary and adaptive resistance to immunotherapy in a tumor‐specific manner, necessitates additional investigation. Consequently, further optimization is imperative to devise optimal combinatorial strategies.

### 2.5 Epigenetic regulation on immunotherapy efficacy

Epigenetic regulations, encompassing modifications in DNA methylation, histone acetylation, and histone phosphorylation, are heritable alterations in gene expression.^75^, ^76^, ^77^, ^78^ These regulations have been found to play a significant role in carcinogenesis and antitumor immunity, including the regulation of antigen‐presenting MHC molecules, immune checkpoint molecules, and lymphocyte activation in both tumor cells and lymphocytes.^75^, ^79^ Nevertheless, the precise impact of epigenetic regulations on clinical immunotherapy responses remains elusive.

**FIGURE 2:**
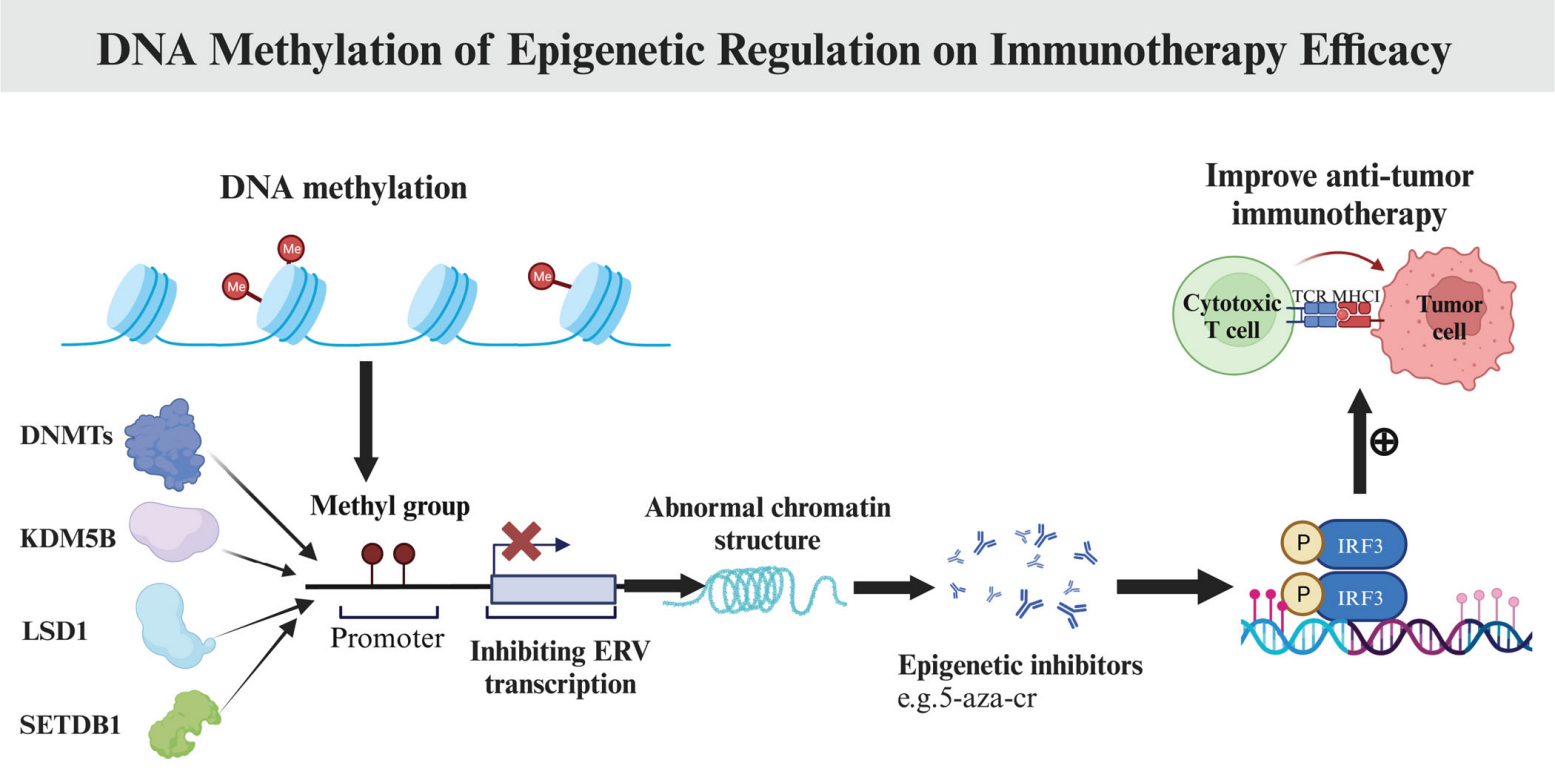
DNA methylation of epigenetic regulation on immunotherapy efficacy. Certain epigenetic modifiers including DNMTs, LSD1, KDM5B, and SETDB1, have been identified as crucial regulators of the antitumor immune response. The epigenic inhibitors such as the DNMT inhibitor 5‐aza‐cr could enhance antitumor immunotherapy. DNMTs, DNA methyltransferases; LSD1, lysine‐specific demethylase 1; KDM5B, lysine demethylase 5B; SETDB1, SET domain bifurcated histone lysine methyltransferase 1; IRF3, interferon regulatory factor 3; TCR, T cell receptor; MHC, major histocompatibility complex; 5‐aza‐cr, 5‐Azacytidine (www.biorender.com).

Several recent studies have identified certain epigenetic modifiers, including DNA methyltransferases (DNMTs), lysine‐specific demethylase 1 (LSD1), Lysine demethylase 5B (KDM5B), and SET domain bifurcated histone lysine methyltransferase 1 (SETDB1), as crucial regulators of the antitumor immune response (Figure 2). In a study conducted by Chiappinelli et al.,^80^ it was demonstrated that the DNMT inhibitor 5‐Azacytidine (5‐aza‐cr) enhanced the sensitivity of nonimmunogenic melanoma cells to anti‐CTLA‐4 therapy and resulted in a reduction in tumor growth in immunocompetent mice. A recent study demonstrated the effectiveness of combined 5‐aza‐cr with pembrolizumab for patients with advanced dMMR CRC, although the activity was limited.^81^ Sheng et al.^82^ found that the absence of LSD1 in vivo resulted in increased H3K4 dimethylation and the derepression of endogenous retroviruses (ERV) transcription, resulting the repressing immunogenic double‐stranded RNAs (dsRNAs). These dsRNAs could trigger an interferon‐dependent antitumor response by activating A‐form dsRNA (A‐RNA)‐sensing proteins including MDA‐5 and PKR5, which could prevent immunotherapy responsiveness.^83^ Subsequent research revealed that the loss of LSD1 led to enhanced T‐cell infiltration and immunogenicity in response to anti‐PD1 therapy in a melanoma mouse model, suggesting the potential for combination therapy utilizing LSD1 inhibitors and anti‐PD‐(L)1.^82^ According to Zhang et al.,^84^ the recruitment of histone 3 lysine 9 (H3K9) methyltransferase SETDB1 by KDM5B plays a mechanistic role in suppressing ERV transcription and antitumor immunity. It is worth noting that SETDB1 is a promising target for enhancing immunotherapy due to its involvement in the KRAB‐associated protein 1 and human silencing hub complex, which regulate immunogenicity across various tumor types.^85^, ^86^ Consequently, the amplification of KDM5B or SETDB1 has been found to be associated with poor OS in patients with renal cell carcinoma treated with nivolumab.^84^, ^85^ In recent studies, the epigenetic regulation of the PD‐1/PD‐L1 pathway has emerged as a potential predictor of immunotherapy response in various malignancies, including NSCLC, melanoma, and GC.^87^, ^88^, ^89^, ^90^ Huang et al.^87^ demonstrated that SETD7 can induce PD‐L1 K162 methylation, thereby inhibiting the interaction between PD‐L1 and PD‐1 in vivo. This mechanism ultimately leads to the suppression of T cell activity and the modulation of tumor immunosurveillance. It was discovered that NSCLC patients who exhibited resistance to anti‐PD‐L1 demonstrated decreased levels of LSD2 and PD‐L1 expression, as well as increased PD‐L1 K162 methylation and SETD7 expression.^87^ These findings provide support for the presence of immunosuppression and evasion mechanisms in cancers characterized by PD‐L1 hypermethylation.^87^ Notably, Qing et al.^89^ observed that anti‐PD‐1 therapy did not enhance T cell inhibition of GC cells expressing PD‐L1P146R, both in vitro and in vivo. Additionally, recent research has indicated that the PD‐1/PD‐L1 colocation scores possess a strong predictive value for the response to anti‐PD‐1/PD‐L1 therapy.^89^, ^91^

Based on aforementioned discoveries, the utilization of epigenetic therapy presents a promising avenue for enhancing the immunogenicity of existing immunotherapies. However, additional investigations elucidating the underlying mechanisms of epigenetic‐induced immunological changes are imperative to broaden the scope of immunotherapy's potential applications.

## 3 CIRCULATING IMMUNE CELLS IN ANTITUMOR IMMUNOTHERAPY

**FIGURE 3:**
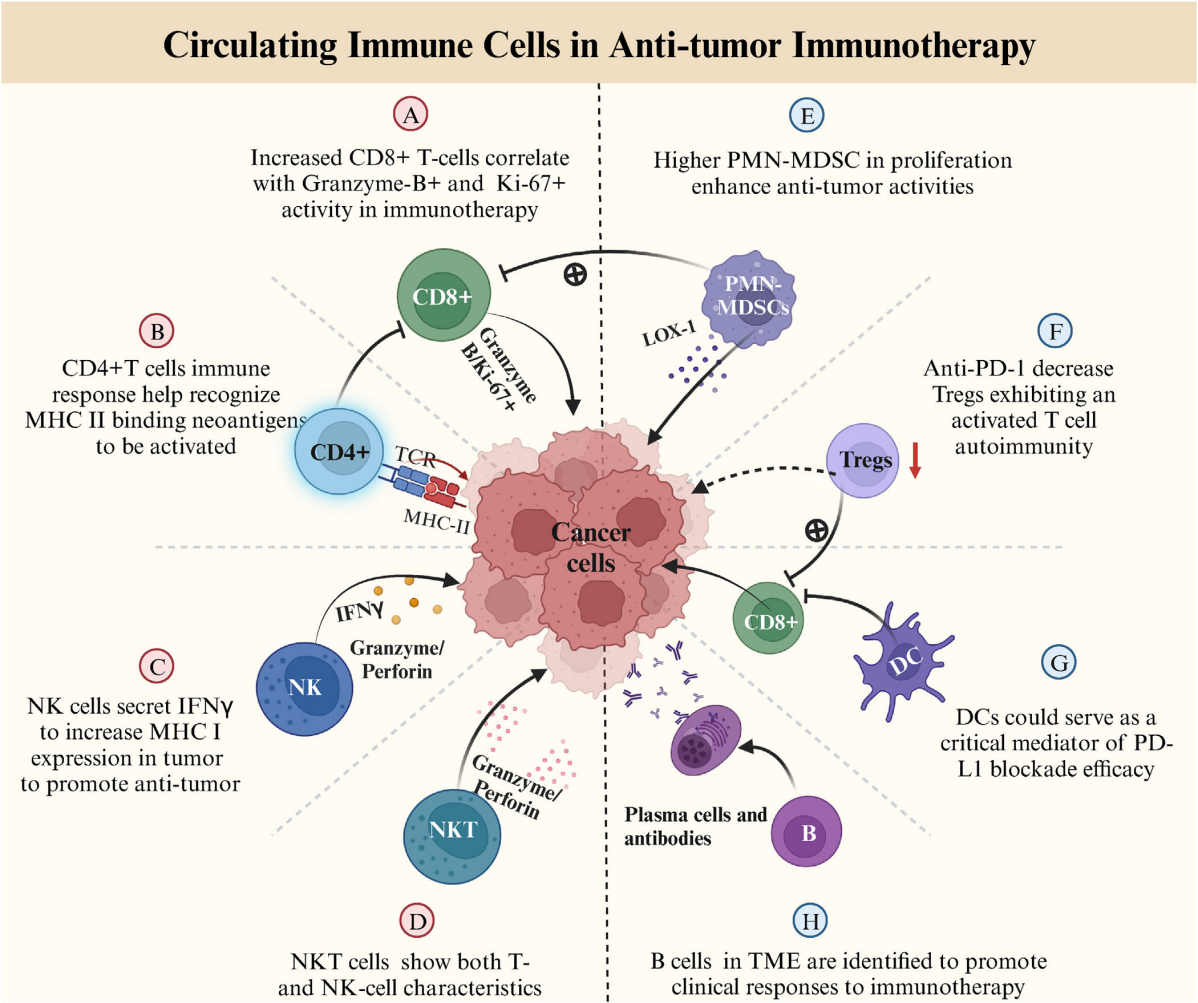
Circulating immune cells biomarkers of response for antitumor immunotherapy. Peripheral blood immune cells (such as CD8+, CD4+, NK, NKT, B, Tregs, DCs, and MDSC cells) have been identified as potential biomarkers in immunotherapy. PD‐L1, programmed cell death ligand‐1; HLA, human leukocyte antigen; NK cells, natural killer cells; DCs, classical dendritic cells; MDSCs, myeloid‐derived suppressor cells; Tregs, regulatory T cells (www.biorender.com).

A large number of studies have recently highlighted peripheral immune system plays a significant role in antitumor therapy, particularly immunotherapy.^92^ Thus, it is of great importance to learn how to boost immune responses against cancer through peripheral immune system. Cancer attacks the hematopoietic system, resulting in the differentiation of hematopoietic stem cells into monocytes and granulocyte in the periphery, which could suppress local immunity after entering TME. The impact of this significant change may lead to other changes in peripheral immunity, leading to systemic disorders of the immune system as a whole, including CD4+ T, CD8+ T cell, myeloid‐derived suppressor cells (MDSCs) proliferation, classical dendritic cells (cDCs) reduction, Treg cells increased, natural killer (NK) and B cell differentiation, abnormal function, and so on. In general, growing evidence suggests that the systemic immune system is developing into a state of inhibitory activity under tumor influence, showing an increase in anti‐inflammatory cells and a decline in pivotal mediators of antitumor immune cells (Figure 3 and Table 3).

**TABLE 3:** Main studies of circulating immune cells to predict immunotherapy in the last 5 years.

Biomarkers	Cancer type	Number of patients	Therapy	Key findings	References
CD8+, CD4+ T lymphocytes					
Cytotoxic CD8+ effector clones	Melanoma	131	Anti‐PD‐1/PD‐L1 or anti‐CTLA‐4	Individuals with fewer expanded CD8+ T cells and lower cytotoxicity had a shorter PFS compared with those with larger clones.	Watson et al.^94^
CD8+PD‐1+TILs	NSCLC	21	Anti‐PD‐1 therapy	PD‐1+ TILs strongly predicted response to ICIs, and correlated with increased OS and durable responses.	Thommen et al.^95^
CCR7‐CD45RA‐CD8+ T cells; TIGIT+PD‐1+CD8+ T cells	NSCLC	263	Anti‐PD‐1 therapy	Lower frequency of peripheral blood CCR7‐CD45RA‐CD8+ T cells and higher TIGIT+PD‐1+CD8+ T cells were correlated with hyper‐progression disease and inferior survival.	Kim et al.^96^
PD‐1+ CD8 T‐cell	NSCLC	29	anti‐PD‐1 therapy	After 4 weeks of treatment initiation, PD‐1+CD8 T‐cell responses were observed in 80% patients exhibiting clinical benefit, whereas 70% exhibiting progression did not display a T‐cell response.	Kamphorst et al.^97^
CD8 T cell, ctDNA	NSCLC	99	Anti‐PD‐1 therapy	Pretreatment peripheral CD8 T cell, ctDNA, and dynamic ctDNA levels are associated with DCB.	Nabet et al.^98^
NK cells and NKT‐like cells					
CD8+T cells, CD4+T cells	Melanoma	20	Anti‐PD‐1 therapy	Responding patients had higher numbers of infiltrating CD4+T cells and CD8+T cells.	Krieg et al.^99^
CD3+, CD4+, and CD8+ T cells, NK cells, CD8+PD1+ Eomes+T cells	NSCLC	74	Anti‐PD‐1 therapy	Longer OS had higher pretreatment CD3+, CD4+, and CD8+ T cells but lower NK cells. The pretreatment CD8+PD1+Eomes+T cells was significantly lower in controlled disease.	Ottonello et al.^100^
PD‐1+CD8 (CD28+CD27‐CD45RO+, TEEM)	NSCLC	77	Anti‐PD‐1 therapy	PD‐1+CD8 TEEM cells exhibited early responses after anti‐PD‐1 therapy and was associated with prolonged PFS and DCB.	Khanniche et al.^101^
PD‐1+CD8+ cells, PD‐1/CD8+ ratio	NSCLC	31	Anti‐PD‐1 therapy	High circulating NK and PD‐1+CD8+ cells combined with low PD‐1/CD8+ ratio in TILs provide a significantly prolonged PFS.	Mazzaschi et al.^102^
PD‐1+CD8+ T‐cells	GC	350	Anti‐PD‐1 therapy	Increased PD‐1+CD8+ T‐cells was prognostic for improved OS and highly correlated with Granzyme‐B+ and proliferative Ki‐67+ activity.	Choo et al.^104^
CD8 + TILs	NSCLC	366	Anti‐PD‐1 therapy	CD8 + TILs as a powerful predictor for PFS and OS.	Hashemi et al.^105^
CD8+CD28‐CD57+KLRG1+T cells	NSCLC	83	Anti‐PD‐1/PD‐L1 or anti‐CTLA‐4	Higher baseline proportion of peripheral senescent CD8+CD28–CD57+KLRG1+T cells were associated with poor ORR, PFS, and OS	Ferrara et al.^109^
CD4+ T‐cell meta‐cluster	NSCLC	60	Anti‐PD‐1 therapy	CD4+ T‐cell meta‐cluster (CXCR3+CCR4−CCR6+ and CXCR3−CCR4−CCR6+ cells) were observed to be significantly correlated with PFS and OS.	Kagamu et al.^115^
CD4+ PD‐1+ T cells	NSCLC	19	Anti‐PD‐1 therapy	High peripheral CD4+ PD1+ T cells could predict longer PFS.	Inomata et al.^116^
CD4+T cells and CD4+/CD8+ ratio	dMMR mCRC	41	Anti‐PD‐1 therapy	Low levels of peripheral CD4+T cells and CD4+/CD8+ ratio could be as positive independent biomarkers for PFS and OS.	Cheng et al.^117^
PD‐1+CD56+ T‐cells	Melanoma	75	Anti‐PD‐1 therapy	Lower median frequency of PD‐1+CD56+ T‐cells was associated with superior OS and PFS.	Bochem et al.^119^
NK cell‐to‐Lox‐1+PMN‐MDSC ratio	NSCLC	62	Anti‐PD‐1 therapy	Higher NK cell‐to‐Lox‐1+PMN‐MDSC ratio was associated with responders, ORR, PFS and OS.	Youn et al.^122^
CD56+CD16‐PD‐1+ NK cells	NSCLC	55	Anti‐PD‐1/PD‐L1 or anti‐CTLA‐4	CD56+CD16‐PD‐1+ NK cells showing good predictive ability to OS.	Gascon‐Ruiz et al.^123^
CD3+CD56+ NKT‐like cells	HCC	25	SBRT	Higher percentage of CD3+CD56+ NKT‐like cells was associated with higher OS.	Li et al.^131^
CD16+ NKT‐like cells	CRC	87	Surgery	CD16+ NKT‐like cells were associated with shorter DFS.	Krijgsman et al.^132^
CD3+CD56+NKT‐like cells	HCC	52	Anti‐PD‐1 therapy	CD3+CD56+NKT‐like cells were exhausted in HCC, while could be improved by PD‐1 blockade	Tao et al.^134^
B lymphocyte subsets					
Memory B‐cell signature	RCC, melanoma	95	Anti‐PD‐1/PD‐L1 or anti‐CTLA‐4	Memory B‐cell signature that was significantly elevated showing clinical benefit	Varn et al.^138^
Intratumoral B cells	NSCLC	891	Anti‐PD‐1 therapy	There was a strong correlation between intratumoral B cells especially plasma cells and longer OS.	Patil et al.^140^
IgM+ memory B cells	NSCLC	150	Anti‐PD‐1 therapy	High levels percentage of peripheral IgM+ memory B cells were associated with longer PFS.	Xia et al.^142^
Other immune cells subsets (DCs, Treg, MDSCs)					
DCs vaccine combined anti‐CD38CpG	Lewis lung carcinoma cells, Mice	11	Anti‐PD‐1/PD‐L1 or anti‐CTLA‐4	Neoantigen DCs vaccine combined with anti‐CD38 and CpG, could produce antitumor immunity against ICIs‐resistant mouse lung cancer cell lines.	Sun et al.^148^
PD‐1+CD4+ Treg	Breast cancer	8	Anti‐PD‐1 therapy	PD‐1 expression on circulating CD4+ Treg in PBC patients is reduced effectively by pembrolizumab.	Toor et al.^156^
Lox‐1+PMN‐MDSCs,Tregs/Lox‐1+PMN‐MDSCs ratio	NSCLC	34, 29	Anti‐PD‐1 therapy	Posttreatment Lox‐1+PMN‐MDSCs were diminished in responders. Tregs/Lox‐1+PMN‐MDSCs ratio≥0.39 had longer median PFS.	Kim et al.^164^

ICIs, immune checkpoint inhibitors; PD‐1, programmed cell death protein‐1; PD‐L1, programmed cell death‐ligand 1; CTLA‐4, cytotoxic T‐lymphocyte antigen‐4; NSCLC, non‐small cell lung cancer; dMMR, deficient mismatch repair; MCRC, metastatic colorectal cancers; HCC, hepatocellular carcinoma; CRC, colorectal cancer; GC, gastric cancer; RCC, renal cell carcinoma; TIL, tumor infiltrating lymphocyte; OS, overall survival; PFS, progression‐free survival; DCB, durable clinical benefit; ORR, objective response rate; NK cells, natural killer cells; HLA, human leukocyte antigen; KLRG1, killer cell lectin‐like receptor G1; SBRT, stereotactic body radiation therapy; MDSCs, myeloid‐derived suppressor cells; PMN‐MDSCs, granulocytic/polymorphonuclear MDSCs; M‐MDSCs, monocytic MDSCs; Treg, regulatory T; cDCs, classical dendritic cells.

### 3.1 CD8+ T lymphocytes

It is well established that peripheral clonal T‐cell responses to ICI are critical determinants for cancer patients since they play a direct role in tumor cells destruction and may enable prognosis.^93^ PD‐L1 and tumor‐infiltrating lymphocytes (TILs) are major players in the ICIs mechanism of action in multiple malignancies. It has been reported that PD‐1+ CD8+ subset represents an exhausted T cell phenotype, which could be targeted to improve survival outcomes in ICIs^94^, ^95^ (Table 3). According to Kim and colleagues, poorer therapy outcomes and high risk of HPD were related to a lower effector memory CD8+ T cell count (CCR7− CD45RA−) at baseline, as well as a higher baseline TIGIT+PD‐1+CD8+ T cells.^96^ These results were evaluated in other tumor types such as NSCLC and melanoma,^96^, ^97^, ^98^, ^99^, ^100^ showing that early increase of circulating PD‐1+CD8+ T cells could be able to predict immunotherapy response. In addition, in the initial PD‐1 treatment, the differentiation state of peripheral CD8+ T cells partially regulates the expansion of TEEM to target antitumor immunity, thus regulating the response of PD‐1 targeted immunotherapy in advanced NSCLC. Recent work from Khanniche et al.^101^ indicates that PD‐1+CD8+T_EEM_/PD‐1+CD8+T_E_ ratio at baseline could be novel indicator to predict the response to ICIs in advanced NSCLC. What is more, PD‐1+ CD8 T cells that express Ki67 have been reported a predictive character of proliferating T cells for the response to anti‐PD‐1 therapy in melanoma and NSCLC.^102^, ^103^ This result is consistent with a most recent study from discovery cohort of 350 GCs patients, indicating that increased PD‐1+CD8+ T‐cells were statistically prognostic for OS and correlated with Granzyme‐B+ and proliferative Ki‐67+ activity with immunotherapy.^104^ Additionally, According to Hashemi et al.,^105^ stromal CD8 + TILs could be powerful predictors of better outcomes in NSCLC patients previously treated with anti‐PD‐1 therapy, based on real‐world study. Consistent with these findings, CD8 TILs were shown to correlate significantly with ORR and PFS in a preliminary study of 38 NSCLC patients treated with pembrolizumab. These outcomes, however, were not influenced by CD4 TILs,^106^ while larger cohort research is needed. Moreover, previous studies also indicate that cell‐intrinsic CD28 signaling in CD8+ T cells could be another mechanism of PD1 blockade efficacy, providing necessary costimulation for naive T cell priming.^107^, ^108^ In line with this finding, patients with a high baseline proportion of peripheral senescent CD8+CD28–CD57+KLRG1+T cells were correlated with poor prognosis to ICIs in NSCLC.^109^

Despite this, most analyses ignore CD8+ T cell phenotypes, thus leaving unresolved the determinants of ICIs activity across different subsets of CD8+ cells. Researchers have shown that patients who respond to ICIs have clones representing circulating and tumor cells enriched for effector markers.^110^ This highlighted that cytotoxic clone persisted throughout multiple ICI cycles, in agreement with previous reports linking cytotoxicity and PD‐1 blockade in NSCLC.^111^ In keeping with this, Watson et al.^94^ assessed transcriptomic responses of single peripheral CD8+ T cells to ICIs. This study showed that ICIs treatment was less effective in individuals with low cytotoxicity and decreased expanded CD8+ T cell clones.^94^ It appears that peripheral CD8+ T cell repertoires pretreatment determines the prognosis of melanoma patients treated with ICIs. Thus, the monitoring of T‐cell subpopulation could provide important information about the clinical outcome of ICIs treatment. However, further research is required to determine the practicality of the method as a predictor of effectiveness.

### 3.2 CD4+ T lymphocytes

Although immunotherapy primarily focused on revitalizing responses by CD8+ T cells, evidence suggests that CD4+ T cells could also influence efficacy differentially^112^ (Table 3). Alspach et al.^113^ confirmed that antitumor immunity would not be induced unless CD4+ T cells immune response helped recognize MHC class II binding neoantigens to be activated. CD4+ T cells have been indicated to play a critical role in the antitumor of CD8+effector T cells.^114^ It is essential that CD4+ and CD8+ T cells work together to activate the antitumor immune response, which is a crucial factor in the response to ICIs.^114^ Further research published in nature indicated that during the treatment of HER2/CD3/C28 tri‐specific antibody targeting HER2 and T cells, CD4+T cells can not only promote the activity of CD8+ T cells but inhibit the division of tumor cells in a cell‐dependent manner. It proved that CD4+ T‐cell immunity plays a major role in antitumor immunity through the activity of CD8+ T cells against cancer cells. Most recently, Kagamu et al.^115^ demonstrated that circulating CD4+ T‐cell subsets (consisting of CXCR3−CCR4−CCR6+ as well CXCR3+CCR4−CCR6+ cells) were observed to be significantly correlated with PFS and OS of patients with NSCLC receiving anti‐PD‐1 therapy, as well as, correlated with the infiltration of CD4+ T cell in TME. This highlights CD4+ T cell meta‐cluster contributes to being potential prediction of immunotherapy. It was also found that high numbers of peripheral CD4+PD1+ T cells could predict higher PFS in NSCLC with anti‐PD‐1 therapy.^116^ Moreover, another multicenter cohort study in dMMR mCRC patients illustrates that low levels of peripheral CD4+T cells and CD4+/CD8+ ratio could be positive independent potential biomarkers for predicting survival outcomes,^117^ further supporting the critical role of CD4+T cells in promoting CD8+ T cells antitumor activities. And by a recent clinicopathological study indicated CD4 in the stroma was consistently associated with PD‐L1 in 50% of the patients; however, the CD8 in intratumoral lesions was associated with PD‐L1 in 1−49%.^118^ Despite this, it is unclear if CD4+ T cells with PD‐L1 expression correlate with prognosis after first‐line anti‐PD‐1 treatment. Taking these findings together, CD4+ T cells are responsible for antitumor immunity. However, latest studies are more focused on evaluating the specific subpopulation contribution to the response of immunotherapy, given that long‐term prognosis has not yet been established prospectively.

### 3.3 NK cells and NKT‐like cells

Another component of antitumor immunity is NK cells, which are capable of directly killing tumor cells and affecting the functions of other immune cells that are antitumorigenic. NK cells can express CD3 as well as CD56, and induce the recruitment of cDC1 cells into TME for controlling tumor immunity. Several studies^99^, ^119^ have identified CD56 associated with NK and NKT cells as a marker to be upregulated in circulation of melanoma patients who respond to anti‐PD‐1 therapy, further suggesting that a myeloid population in peripheral blood may be a good predictor to identify most responsive patients (Table 3). To date, NK cells have been proven to express T cell immune checkpoints PD‐1, CTLA4, lymphocyte activation gene 3 protein, and their ligands could be a key role in dampening NK antitumor responses. Thus, blocking this interaction with ICIs could enhance NK cell activity. Moreover, NK cells secret IFNγ to increase MHC class I expression in tumor cells and, thus, enhance their vulnerability to T cells which could be magnified with checkpoint inhibitors.^120^, ^121^ Just recently, studies have shown that anti‐PD‐1 treatment leads to improved outcomes in patients with NSCLC who have increased peripheral NK cells.^122^ Growing studies have demonstrated that lymphocytes and NK cells are key roles in immune defense against tumor cells. A recent prospective study has identified a subset of circulating CD56+CD16‐PD‐1+ NK cells showing good predictive ability to ICIs response in NSCLC patients with immunotherapy.^123^ In addition, considering cancer patients commonly inhibit deficiency in NK cells, growing evidence has indicated that adoptive transfer of NK cells could be a useful immunotherapy tool across a number of solid cancers.^124^, ^125^, ^126^ And beyond immunotherapy, researchers suggest that molecularly targeted agents can elicit NK cell‐mediated antitumor immunity, providing further evidence that NK cells will play an increasingly key role in cancer treatment.^126^ Despite this, there are still limited data on how well NK cells predict PFS or OS in cancer patients receiving ICIs.

NKT cells are unique subgroups of lymphocytes that show both T‐ and NK‐cell characteristics. There are two types of NKT cells: Type I or TCR‐restricted invariant cells, and TCR‐diverse type II. Additionally, “NKT‐like” cells have been identified as a third group that resembles NKT cells. As a poorly understood, controversial, and heterogeneous lymphocyte population, NKT‐like cells play a major role in innate as well as adaptive immunity.^127^, ^128^ Coexpression of CD3 and CD56 cell markers was used to define NKT‐like cells,^129^ further classified as CD3+CD56+CD16+/− and CD3+CD56−CD16+ cells.^130^ As opposed to classical NKT cells, NKT‐like cells are activated via HLA‐associated antigen presentation, not CD1d.^130^ Recent studies on different types of cancer have highlighted the importance of NKT‐like cells. A higher percentage of NKT‐like cells was associated with superior OS in patients with HCC receiving stereotactic body radiotherapy.^131^ In colon carcinoma patients treated with surgery alone, preoperative CD3+CD56+NKT‐like cells were increased in number, and the percentage of CD16+ NKT‐like cells was correlated with poor disease‐free survival.^132^ In GC, lower CD3+CD56+ NKT‐like cells inhibited worse survival.^133^ Moreover, Tao et al.^134^ discovered that CD3+CD56+NKT‐like cells were functionally exhausted in HCC, which could be effectively improved by PD‐1 blockade. The results of these studies inspired us to look at CD3+CD56+NKT‐like cells as potential immunotherapy targets. However, possible prediction of NKT‐like cells for clinical outcomes in cancer treated with ICIs remains unknown so far.

### 3.4 B lymphocytes

It has been extensively reviewed in the literature that T cells perform a key role in ICIs therapy, while B cells have also been implicated. Recently, B cells and tertiary lymphoid structures (TLSs) in TME were identified to promote the clinical responses to ICIs.^135^, ^136^ Patients with ICIs monotherapy showed a modest increase in circulating memory B cells, CD21lo B cells, and plasma blasts.^137^ Besides, memory B‐cells in gene‐expression profiling were significantly elevated in patients receiving ICIs therapy for urothelial carcinoma (UCC) and melanoma^138^ (Table 3). Typically, B cells are identified by CD19 and CD20. CD19, a classic marker for B‐cell identification, has been shown to play a key role in B‐cell activation and proliferation. A higher level of this marker may result in improved clinical outcomes due to increased humoral response.^139^ Evidence suggests that B cells play a key role in immunotherapy responses. This is particularly the case given the most recent data from Patil et al.^140^ showed that there was a significant correlation between intratumoral B cells, especially plasma cells, and the longer OS treated with atelizumab in NSCLC patients. This finding not only determines the prognostic role of intratumoral B cells in regard to immunotherapy response but also proves that plasma cells also exist in TLS tissue with potential marker function. Of note, the prediction value of B cells in peripheral blood has also been evaluated. DeFalco et al.^141^ indicated that high plasmablast numbers were seen in patients responding to Ipilimumab. Another research from Liliang Xia et al. found for the first time that high levels percentage of baseline peripheral IgM+ memory B cells were associated with superior PFS in advanced NSCLC patients receiving anti‐PD‐1 treatment.^142^ It turns out that these findings are more prominent than typical T‐cell signatures that are used to guide immunotherapy today. It is important to validate, extend, and apply validate, extend, and apply these intriguing analyses to other cancers amenable to checkpoint blockade as well as to understand the mechanisms that underlie these findings.

### 3.5 Classical dendritic cells

The traditional view shows that therapeutic effect of ICIs is correlated to PD‐L1 expression in tumor cells. In fact, aside from tumor cells, the majority of cells that express PD‐L1 are antigen‐presenting cells, including macrophages and, at even higher levels, cDCs, which have been considered to correlate with ICIs response in different cancer types^4^, ^143^, ^144^, ^145^ (Table 3). It is shown that if PD‐L1 expression in cDCs rather than macrophages are targeted depletion, but not macrophages, the response of CD8+T cells and PD‐L1 inhibitors will be greatly reduced.^4^, ^93^, ^143^, ^144^ This evidence suggests cDCs could serve as a critical mediator of PD‐L1 blockade efficacy. Moreover, further evidence from Kathryn E. Yost et al.^146^ demonstrated that cDCs can transport tumor antigens from the TME to the peripheral tumor‐draining lymph node, and stimulate the production of antigen‐specific T cells. Then activated T cells circulate in the peripheral blood and are transported to the tumor where they kill cancer cells, highlighting that cDCs are linked to tumor‐resident and tumor‐extrinsic T cell immune response.^93^, ^147^ Increasing evidence suggests DCs could be crucial in initiating antitumor immunity. Therefore, improving DC numbers and status is important for suppressing tumor growth. It has been reported that the application of neoantigen dendritic cell vaccine combining anti‐CD38 and CpG, which induces antitumor immunity in mice with tumors resistant to ICIs and further develops DC vaccine therapy.^148^ Trials are ongoing to determine whether cDCs are appropriate immunotherapy biomarkers.

### 3.6 Treg cells

As Tregs are known to be crucial in immune homeostasis and autoimmunity prevention, a decrease in Treg function may trigger overreactive autoimmune responses and disorders.^149^, ^150^ Tregs have been shown to increase in periphery and TIL of patients with NSCLC, breast cancer, colorectal cancer, and other malignancies.^4^, ^151^ Growing evidence demonstrates that Treg promotes tumor immune escape through different mechanisms. PD‐1, PD‐L1, and CTLA‐4 are also over expressed on PD‐1/PD‐L1‐producing Tregs in the TME, which transmit inhibitory signals.^149^, ^152^ It is noteworthy that PD‐1/PD‐L1 pathway is one of the important mechanisms by which Treg inhibits T cell activity and thus exerts its immunosuppressive function.^149^, ^152^, ^153^ Depletion of Tregs in tumors would be effective in treating and preventing tumor progress in ICIs. Thus, ICI therapies will be more effective if Tregs are considered (Table 3). In the clinic, Kamada et al.^15^ indicate that PD‐1 blockade significantly increased the number of suppressive PD‐1+ effector Treg cells in HPDs advanced GC, thereby modulating T cell autoimmunity. This pattern has also been described by other authors.^154^ Subsequent studies have identified that PD‐1‐deficient Tregs exhibit an activated phenotype and enhance immunosuppressive functions linked to reduced PI3K‐AKT signaling.^155^ In vivo, Toor et al.^156^ found that pembrolizumab can inhibit the expression of FOXP3 on Treg's surface, thereby weakening Treg's immunosuppressive function in melanoma patients. Taking these findings together, combining anti‐CTLA‐4 with nivolumab statistically enhanced the efficacy of patients with metastatic cancers, and may be even more effective than monotherapies.^157^, ^158^, ^159^ However, it is still unclear how the specific mechanism of action works. Research is needed to further clarify how ICIs affect Treg in a dual and complex way.

### 3.7 Myeloid‐derived suppressor cells

MDSCs are a class of highly heterogeneous cells of immature myeloid progenitors, that induce the dysfunction of hematopoietic stem cells, and suppress antitumor immunity of T and NK cells when progressed.^160^, ^161^ MDSCs can be further clarified into two subtypes: granulocytic/polymorphonuclear (PMN‐MDSCs) and monocytic MDSCs (M‐MDSCs).^162^ Concerning PD‐1/PD‐L1 inhibitors, data from Gao et al.^161^ have demonstrated that immunotherapy is currently characterized by drug resistance because of immunosuppressive cells within TME, where MDSC‐mediated immunosuppression may play a role (Table 3). Patients with NSCLC have a high rate of PMN‐MDSCs, which may make a promising strategy to overcome immunotherapeutic resistance.^163^ Regarding NSCLC, two prospective cohorts pooled analysis from Kim et al.^164^ identified that Lox‐1+PMN‐MDSCs were diminished in responders and inversely correlated with the percentage of Lox‐1+PMN‐MDSC after one cycle of nivolumab, while no significant difference was observed at baseline. Interestingly, the authors further evaluated Tregs/Lox‐1+PMN‐MDSCs ratio (≥0.39) had statistically longer median PFS, highlighting that higher PMN‐MDSC in proliferation might be related to poor outcomes of ICIs therapy. Moreover, another study indicated that NK/Lox‐1+PMN‐MDSCs ratio was strongly correlated with clinical outcomes in patients with NSCLC after one cycle of nivolumab, further proving the accumulation of MDSCs may lead to poor prognosis in immunotherapy.^122^ There is, however, a lack of prognostic significance of MDSCs in various types of cancers, and further validation is necessary.

## 4 SOLUBLE SYSTEMIC MARKERS OF IMMUNOTHERAPY

Several serum‐based biomarkers have been extensively studied or are presently being investigated. In the context of anticancer immunotherapy, numerous systemic circulating markers have been identified as potential prognostic indicators, including soluble PD‐L1 (sPD‐L1), blood‐based TMB (bTMB), and ctDNA. Additionally, other routine circulating markers have also demonstrated significant relevance in the longitudinal assessment of clinical outcomes during immunotherapy (Figure 1).

### 4.1 Soluble PD‐L1 expression

Apart from tumor tissue biomarkers, soluble form of PD‐L1 can also be detectable in the bloodstream.^165^ The expression of sPD‐L1 has also been reported to occur during maturation from antigen‐presenting cells such as MDSCs when cytokines are present.^166^ Moreover, the increased sPD‐1 levels in cancer cells are also associated with absent TILs, avoiding the inhibition of T cells in antitumor activity.^167^, ^168^, ^169^ In cancer patients, sPD‐L1 was found in high concentrations, possibly contributing to immunosuppression or resistance to ICIs.^165^, ^170^ In fact, it has been reported that sPD‐L1 is a possible predictor of survival and that high sPD‐L1 levels adversely affect survival in a variety of cancers^13^, ^170^ (Table 4). In particular, a higher level of sPD‐L1 prior ICIs treatment (anti‐CTLA‐4/anti‐PD‐1 blockade), was more likely to suffer from progressive diseases and poorer clinical outcomes in different types of tumors, such as melanoma, NSCLC, breast cancer, and other solid tumors.^166^, ^168^, ^171^, ^172^, ^173^ One hypothesis is that sPD‐L1 may result in an increase in tumor burden, aberrant splicing, or amplified immune exhaustion in tumor cells. Several studies from Oh et al.^174^ and Mahoney et al.^175^ also reported a dynamic change in the levels of sPD‐L1 negatively correlated with ICIs therapy. On the basis of these results, the sPD‐L1 levels were mostly tested at baseline and some of them were retrospective while a quite few dynamic analyses were carried out in the majority of patients. Future prospective studies are needed. Moreover, in different studies, sPD‐L1 cut‐off values varied widely, contributing to heterogeneity.^176^ As well, previous molecular signatures and therapies from different types of cancer could affect sPDL‐1 release.^177^ Thus, future prospective studies using standard assessment methods should be performed to determine and validate the optimal cut‐off value as well as prognostic value regarding immunotherapy efficacy in different solid tumor settings.

**TABLE 4:** Main studies of solute systemic biomarkers to predict immunotherapy in the last 5 years.

Cancer type	Number of patients	Therapy	Key findings	References
sPD‐L1				
NSCLC	15	anti‐PD‐L1 therapy	Significant increase in CD8‐ and sPD‐1‐positive cells was observed in the Murine Carcinoma 38/mPD‐L1 tumor lesion after treatment.	Gong et al.^170^
NSCLC	87	Nivolumab	In two cycles of nivolumab, higher or stable sPD‐1 levels were independently associated with longer PFS.	Tiako Meyo et al.^171^
Breast cancer	86	Anti‐PD‐L1 therapy	Patients with sPD‐L1 ≥ 8.774 ng/ml exhibited significantly worse PFS and OS than those with sPD‐L1 < 8.774 ng/mL .	Han et al.^172^
NSCLC	43	Nivolumab	High sPD‐L1 and low Granzyme B were associated with poor PFS and OS.	Costantini et al.^173^
8 solid tumors^a^	128	Anti‐PD‐1/PD‐L1 or anti‐CTLA‐4	Pretreatment sPD‐L1 levels were significant predictors of PFS and OS	Kim et al.^174^
RCC, melanoma	169	Nivolumab	Patients with PD or SD had an increase in sPD‐L1, while those with ORR did not have an increase.	Mahoney et al.^175^
bTMB				
NSCLC	1,118	Durvalumab + tremelimumab	A validation set of bTMB the 20 mut/Mb showed a favorable median OS.	Si et al.^181^
NSCLC	6507	Atezolizumab	PFS and OS both increased in the bTMB ≥ 16 mut/Mb. bTMB ≥ 13.6 mut/Mb by F1L CDx assay indicated superior PFS.	Peters et al.^184^
NSCLC	50	Anti‐PD‐1/anti‐PD‐L1 therapy	bTMB of ≥6 mut/Mb was correlated with better PFS and ORR.	Wang et al.^185^
NSCLC	169	Atezolizumab	bTMB ≥ 16 mut/Mb was correlated with better ORR.	Kim et al.^187^
NSCLC	809	Durvalumab + tremelimumab	The OS of those with a bTMB of 20 mut/Mb was improved .	Rizvi et al.^188^
NSCLC	853	Atezolizumab	ctDNA‐adjusted bTMB by using an upside‐down U‐shaped curve was significant associated with OS and PFS in OAK and POPLAR cohort.	Nie et al.^189^
ctDNA				
NSCLC	99	Anti‐PD1 therapy	Pretreatment ctDNA could be as a predictive role in identifying patients with DCB.	Nabet et al.^98^
Melanoma	70	Anti‐PD1 therapy	Significant association was observed between presence of ctDNA in PD patients.	Warburton et al. 2020)^192^
Melanoma	33	Anti‐PD‐1/PD‐L1 or anti‐CTLA‐4	Comparing undetectable ctDNAs with detectable ctDNAs, patients with undetectable ctDNAs had significantly longer PFS.	Pedersen et al.^193^
Pan‐tumor	1149	Durvalumab	On‐treatment reductions of ctDNA was correlated longer PFS, OS, and ORR.	Zhang et al.^196^
NSCLC	31	Anti‐PD‐1/PD‐L1 therapy	As part of surveillance, ctDNA can be highly sensitively used to detect minimal residual disease and predict risk of eventual progression.	Hellmann et al.^197^
Routine circulating biomarkers				
NSCLC	134	nivolumab	Lower baseline ANC, higher ALC, and higher AEC were associated with superior PFS and OS.	Tanizaki et al.^201^
RCC	73	Nivolumab	Lower CRP had significantly longer PFS, while no correlation with OS.	Takamatsu et al.^203^
RCC	41	Nivolumab + ipilimumab	Normal (CRP remains < 1.0 mg/dL) and normalized (CRP decreased < 1.0 mg/dL) CRP inhibited significant correlation with PFS.	Ishihara et al.^204^
Melanoma	140	Nivolumab + ipilimumab	High IL‐6, CRP(> ULN) and the N/L ratio are prognostic predictors associated with poor OS.	Laino et al.^205^
NSCLC	187	Nivolumab	NLR < 5 were associated with PFS and OS, but not of ORR or DCR.	Russo et al.^207^
NSCLC	79	Anti‐PD1 therapy	baseline NLR (2.43) and preoperative NLR (1.48) were correlated with poor pathological response and DFS.	Sun et al.^208^
NSCLC	249	Anti‐PD‐1/PD‐L1 or anti‐CTLA‐4	Patients with high pretreatment NLR (≥5) and low HGB (< 12 g/dL) had a median OS, regardless of PD‐L1 expression.	Ayers et al.^209^
NSCLC	239	Anti‐PD‐1/PD‐L1 or anti‐CTLA‐4	Tumor regression was associated with a decline in NLR of greater than 10% four weeks after ICIs.	Hwang et al.^213^
NSCLC	45	Nivolumab	The combined increase of cfDNA and NLR > 20% on treatment was associated with significantly worse median OS.	Passiglia et al.^214^
NSCLC	466	Anti‐PD‐1/anti‐PD‐L1 therapy	Pretreatment LIPI combining with dNLR (> 3) and LDH (> ULN) were associated with OS.	Mezquita et al.^215^
NSCLC	62	Nivolumab	Patients had higher incidences of thrombocytosis, neutrophilia, PLR levels, and dNLR levels than the general population as a whole.	Russo et al.^218^
Pancreatic cancer	122	Anti‐PD‐1/PD‐L1 or anti‐CTLA‐4	Elevated baseline SII (≥566) and ΔNLR% (≥−0.1) were significantly correlated with an increased risk of death.	Shang et al.^219^
NSCLC	102	Anti‐PD‐1/PD‐L1 or anti‐CTLA‐4	Only PNI (≥45.5) but not NLR or CRP showed a trend towards being an independent prognostic factor for superior OS.	Shoji et al.^220^
HCC	296	Atezolizumab + bevacizumab	Patients with high NLR (≥5) correlate with shortened OS and PFS. PLR ≥ 300 have worse OS.	Wu et al.^221^

ICIs, immune checkpoint inhibitors; PD‐1, programmed cell death protein‐1; PD‐L1, programmed cell death‐ligand 1; CTLA‐4, cytotoxic T‐lymphocyte antigen‐4; NSCLC, non‐small cell lung cancer; OS, overall survival; PD, progressive disease; SD, stable disease; HCC, hepatocellular carcinoma; PFS, progression‐free survival; DCB, durable clinical benefit; ORR, objective response rate; DCR, disease control rate; sPD‐L1, soluble PD‐L1; bTMB, blood‐based tumor mutational burden; ctDNA, circulating tumor DNA; cfDNA, circulating free DNA; ULN, upper limit of normality; NLR, neutrophil‐to‐lymphocyte ratio; dNLR, derived‐NLR; AEC, eosinophil count; HGB, hemoglobin; PLR, platelet‐to‐lymphocyte ratio; SII, systemic immune‐inflammation index; PNI, prognostic nutritional index; LIPI, lung immune prognostic index; CRP, C‐reactive protein.

^a^8 solid tumors: NSCLC; melanoma; SCLC, small cell lung cancer; UCC, urothelial carcinoma; RCC, renal cell carcinoma; HNSCC, head and neck squamous cell carcinoma; salivary gland cancer.

### 4.2 Blood‐derived TMB

MSI and TMB derived from blood (bMSI and bTMB) have been evaluated as surrogate markers for tissue‐derived MSI and TMB.^178^, ^179^, ^180^ There are many advantages to detecting bMSI status and bTMB using liquid biopsy collection over tissue analysis, including accessibility and noninvasive nature, as well as capturing tumor heterogeneity that would be lost in tissue analysis.^180^, ^181^ The bMSI correlates well with tissue‐based analyses, allowing us to identify patients who will likely respond to ICIs.^182^, ^183^ In principle, mutations per megabase in bTMB are evaluated as in tissue TMB, with clonal hematopoietic mutations removed to avoid confounding the result. It has been shown that higher levels of bTMB correlate with longer survival following immunotherapy^181^, ^184^, ^185^, ^186^ (Table 4). In the B‐F1RST trial,^187^ bTMB predicted response to atezolizumab using bTMB ≥16 cutoffs. Similarly, bTMB analysis results of the MYSTIC trial that assessed durvalumab and tremelimumab in NSCLC supported the OS benefit of bTMB, However, different threshold was used (≥20 mutations per megabase).^188^ While bTMB has garnered great interest as a possible replacement for tTMB, recent evidence indicates that bTMB cannot reliably predict ICIs survival, especially OS.^179^, ^181^ According to a recently updated report from the phase 2 B‐F1RST trial, both OS and PFS were not predicted by bTMB.^187^ Moreover, Nie et al.^189^ observed a U‐shaped curve between bTMB and survival, showing that patients with low or high bTMB had longer PFS and OS than patients with medium bTMB. Data above indicate that bTMB does not have a good prediction ability when it comes to predicting the clinical benefit of ICIs. Moreover, most data to date are retrospective and there are several panels to determine bTMB. Despite these promising results, standardization and further exploration of bTMB as an ICI predictor are required.

### 4.3 Circulating‐tumor DNA

Recent advancements in liquid biopsy have made ctDNA a sensitive, noninvasive, and accurate method for monitoring ICIs response through dynamic changes in tumor actionable resistance mutations. ctDNA is a portion of cfDNA that originates from tumors. During apoptosis or necrosis, it is possible to sample and analyze ctDNA shed into the bloodstream non‐invasively. By combining digital‐based PCR (d‐PCR) and NGS with bioinformatic analyses, mutant sequences from ctDNA typically found in circulation can be detected in a sensitive and specific manner.^190^, ^191^ Studies have increasingly demonstrated the clinical utility of monitoring ctDNA in ICIs therapy in different types of cancer^192^, ^193^, ^194^, ^195^ (Table 4). As demonstrated by Nabet et al.,^98^ dynamic ctDNA could be an adjunctive tool for identifying NSCLC patients that will receive ICIs that will provide durable clinical benefits (DCB). In this setting, Zhang et al.^196^ also found that on‐treatment ctDNA dynamics also contributed to assessing response in three‐phase I/II trials of ICIs across 16 advanced tumor types. It was demonstrated that reductions in ctDNA variant allele frequencies on treatment correlate with a longer PFS, OS, and ORR following ICIs.^196^ In accordance with the findings of a systematic review from Al‐Showbaki et al.,^194^ ctDNA might provide a real‐time molecular marker for the assessment of ICIs in patients with advanced solid tumors. There is also preliminary evidence that ctDNA changes may play a role in identifying long‐term responders to ICIs who will eventually progress. It has been demonstrated that ctDNA clearance is associated with significantly better outcomes beyond 2 years after initiating ICIs.^197^ Of note, early evidence did not seem to indicate a consistent association between ctDNA dynamic changes and TMB or PD‐L1 expression.^194^, ^198^, ^199^ Compared with this, Kim et al.^200^ observed a correlation between improved ORR, ctDNA reduction, and MSI‐H status in metastatic GC, but did not report PFS data. Whether ctDNA and other markers of response can be combined to improve clinical utility will require further research. According to the landscape of clinical trials, liquid biopsies are not just capable of providing an independent measurement of therapeutic response, but may also decrease the underestimation of ICIs benefits associated with radiographic response criteria. However, the presence of false‐positive mutations in control plasma DNA makes it difficult to detect low‐level true‐positive mutations in ctDNA.^137^ The detection of ctDNA is also uncertain since there is no uniform standard, and the threshold is not defined. Moreover, Statistically, it appears that large cohorts of research are lacking. Differences in sensitivity and discovery range between available assays need to be quantified.

### 4.4 Routine circulating biomarkers

Increasing studies have examined the predictive value of peripheral blood biomarkers in tumor immunotherapy due to their cost‐effectiveness and accessibility (Table 4). Multivariate analysis of NSCLC patients receiving nivolumab shows that the baseline elevated absolute neutrophil count (ANC), lymphocyte count (ALC), and eosinophil count (AEC) were independently related to worse PFS and OS.^201^ The present studies showed that patients with high levels of CRP during treatment correlated with poor clinical outcomes in different solid tumor types.^202^, ^203^, ^204^, ^205^ This may contribute to that inflammation could promote tumor proliferation, metastasis, and adaptive immunity destruction. However, there remains controversy surrounding these results, which differ from other studies claiming that these biomarkers lack predictive power and meaningful baseline values,^206^ suggesting that single peripheral blood parameters alone may not be sufficient for accurately predicting the clinical efficacy of ICIs. There has been some research into the use of composite peripheral parameters as predictors of immunotherapy response. For instance, previous studies showed that increased pretreatment neutrophil‐to‐lymphocyte ratio (NLR) negatively correlates with worse outcomes in different cancer types with ICIs treatment.^207^, ^208^, ^209^, ^210^ NLR, the equilibrium of circulating NEU and LYM, could reflect the balance between inflammation‐mediated and antitumor immunity through the suppression of T cells among different malignancies.^211^, ^212^ Most recent work from Hwang et al.^213^ identifies that increasing intratumorally T cell clones in the periphery are associated with decreased NLR, suggesting that dynamic changes are indirect peripheral measures of antitumor immune response receiving immunotherapy. In this regard, there is also evidence that NLR changes following ICI treatment are negatively associated with prognosis in cancer patients during ICIs treatment.^213^, ^214^ Furthermore, due to its inclusion of monocytes and other granulocyte subpopulations, researchers suggest that the derived‐NLR (dNLR) may have greater relevance to clinical outcomes than the NLR.^215^, ^216^

In addition, when ICIs are used, statistically significant differences in LDH expression are observed in patients with a poor prognosis. This may be due to the tumor‐derived lactic acid accumulating in CD8+ T cells and preventing them from exporting lactate, resulting in metabolic disorders.^217^ Taking dNLR and LDH together, the results of a multicohort study indicated that high dNLR (>3) and LDH (>upper limit of normality [ULN]) at baseline were associated with poor prognostic of ICIs therapy^215^ (Table 4). In this regard, a higher level of lung immune prognostic index (LIPI) score combined with LDH and dNLR was associated with prolonged OS, which can effectively serve as a potential tool to identify patients who could benefit from immunotherapy.^215^ Other investigations also identify platelet‐to‐lymphocyte ratio (PLR), systemic immune‐inflammation index (SII), lymphocyte‐to‐monocyte ratios, and prognostic nutritional index (PNI) were considered as predictors of ICIs response.^218^, ^219^, ^220^, ^221^, ^222^ All of these circulating biomarkers can be performed longitudinally without exhausting tumor samples, enabling early diagnosis. Thus, prospective clinical trials should be conducted to evaluate noninvasive biomarkers. Additionally, we also need to create predictive model that incorporates these different biomarkers, which might aid physicians in guiding routine immunotherapy practice.

## 5 GUT MICROBIOME IN IMMUNOTHERAPY

The function of commensal bacteria plays an important role in maintaining the immune and physiologic homeostasis of the host.^223^ There is increasing evidence that the gut microbiota is a tumor‐extrinsic biomarker for ICIs response^223^ (Table 5). It is well established that the gut microbiota composes a substantial part of the immune response to ICIs especially anti‐PD‐1.^224^, ^225^, ^226^ However, it should also be noted that published microbial signatures that are associated with clinical outcomes have discrepancies. More recently, McCulloch et al.^227^ identified essential gut microbial signatures by evaluating multicohort of melanoma patients to predict the response of anti‐PD1 therapy. This study showed that microbiota composition (Lachnospiraceae/Ruminococcaceae families of Firmicutewas and Actinobacteria phylum) optimally related to clinical outcome following 1 year of initiation of treatment. In contrast, gram‐positive bacteria have been associated with host inflammation, increased NLR levels in peripheral blood, and poor outcomes.^227^ Another largest metagenomic study identified other microbiota species (Akkermansia muciniphila, Bifidobacterium pseudocatenulatum, and Roseburia spp.) were associated with responders, while no one could serve as a consistent biomarker across studies.^228^ Aside from melanoma, mansia muciniphila and Clostridium pathways were associated with favorable responders in epithelial tumors and renal cell cancer respectively, followed by ICIs therapy.^229^, ^230^ Responder patients in NSCLC receiving nivolumab were identified to have abundant Alistipes putredinis, Bifidobacterium longum, and Prevotella copri.^231^ Moreover, patients with HCC were also identified to have a higher proportion of Akkermansia muciniphila and Ruminococcaceae spp. among responders.^232^ Based on these findings, ICI‐mediated immunity may result in the limited concordance between bacterial species identified across studies. In spite of this, the exact microbiota has not been fully determined for all ICIs therapies, particularly for the response to anti‐CTLA4, due to the limited number of patients involved.^224^, ^233^, ^234^ There is a need to clarify the mechanism and reaction of bacterial gene pathways through larger cohort studies.

**TABLE 5:** Main studies of gut microbiome to predict antitumor immunotherapy in the last 5 years.

Cancer type	Microbiome	Therapy	Key findings	References
Melanoma	Lachnospiraceae/Ruminococcaceae families of Firmicutewas and Actinobacteria phylum	Anti‐PD‐1	Microbiota composition optimally related to clinical outcome following 1 year of initiation of treatment.	McCulloch et al.^227^
Melanoma	Akkermansia muciniphila, Bifidobacterium pseudocatenulatum, and Roseburia spp.	Anti‐PD‐1/PD‐L1 or anti‐CTLA‐4	These microbiomes were associated with responders, but none could be considered promising biomarkers.	Lee et al.^228^
Epithelial tumors	Akkermansia muciniphila	Anti‐PD‐1/PD‐L1 or anti‐CTLA‐4	Akkermansia muciniphila were associated with favorable outcomes.	Routy et al.^229^
RCC	Clostridium hathewayi	Nivolumab	Antibiotics use have been found to reduce ORR and markedly facilitating the dominance of distinct species.	Derosa et al.^230^
NSCLC	Alistipes putredinis, Bifidobacterium longum, and Prevotella copri	Nivolumab	Alistipes putredinis, Bifidobacterium longum, and Prevotella copri are associated with responders whereas Ruminococcus_unclassified enriched in nonresponders.	Jin et al.^231^
HCC	Akkermansia muciniphila and Ruminococcaceae.	Anti‐PD‐1	Responders were identified to have a higher proportion of Akkermansia muciniphila and Ruminococcaceae.	Zheng et al.^232^

ICIs, immune checkpoint inhibitors; P PD‐1, programmed cell death protein‐1; PD‐L1, programmed cell death‐ligand 1; CTLA‐4, cytotoxic T‐lymphocyte antigen‐4; NSCLC, non‐small cell lung cancer; HCC, hepatocellular carcinoma; ORR, objective response rate; RCC, renal cell carcinoma.

## 6 CONCLUSION AND PERSPECTIVE

Despite the significant advancements made by immunotherapy in the field of cancer treatment, the efficacy of this approach remains restricted due to the emergence of primary or acquired resistance, as well as the accompanying toxicities. Extensive research endeavors have been undertaken to establish reliable biomarkers capable of identifying patients who are likely to respond positively to ICIs while minimizing adverse effects. However, in order to extend the advantages of ICIs to a broader spectrum of cancer patients, it is imperative to acquire a more comprehensive understanding of the underlying mechanisms governing resistance and toxicity.

Extensive research has been conducted on the impact of host factors, such as tumor genetics and immune and nonimmune components of the TME, on the response to ICIs.^67^ However, despite the potential utility of tissue PD‐L1 expression or TMB as biomarkers for ICIs, the accuracy in predicting treatment efficacy varies across studies due to variations in definitions and thresholds.^235^ Recent advancements in understanding epigenetic and oncogenic mechanisms have revealed their critical involvement in regulating the antitumor response at the interface between cancer cells and T‐cells.^1^, ^75^, ^236^ In contrast, circulating biomarkers have demonstrated potential as indicators of antitumor immunotherapy, yet their utilization in clinical settings remains limited. The investigation of circulating biomarkers, including T cells, B cells, sPD‐L1, MDSCs, and other immune cells, has been insufficient, hindering the comprehensive evaluation of cancer polymorphism and the understanding of both the host's immune status and tumor characteristics. However, the predictive accuracy was compromised by the inherent constraints of utilizing individual markers for response prediction. Consequently, the integration of multiple biomarkers is commonly employed in the prediction procedure. A more systematic approach is needed to fully exploit the informative value of these biomarkers.

In order to optimize the efficacy of immunotherapy, the integration of neural network‐based machine‐learning models with diverse‐omics layers have emerged as a promising strategy to enhance the accuracy of predicting response to ICIs.^237^, ^238^ Computational methodologies that integrate transcriptomic, epigenomic, and genomic data hold great potential in identifying biomarkers associated with response and resistance to ICIs. Furthermore, the development of robust algorithms capable of extracting biologically relevant information will expedite the implementation of reverse translational approaches in this field. Moreover, recent advancements in single‐cell technologies, such as single‐cell RNA sequencing and single‐cell sequencing assay for transposase‐accessible chromatin, have demonstrated their ability to detect previously uncharacterized immune cell populations and novel cellular states, while providing mechanistic insights at a more detailed level. Additionally, the integration of single‐cell technologies with clinical and preclinical investigations, aided by computational algorithms, proves particularly valuable in comprehending immunosuppressive mechanisms, discovering novel biomarkers, unraveling underlying biological processes, and facilitating the development of combinatorial therapeutic approaches.

In conclusion, the existing evidence necessitates further investigation to comprehensively comprehend the individual and collective impact of these factors. Such studies will aid in the creation of innovative diagnostic, prognostic, and therapeutic approaches to overcome the current constraints associated with immunotherapy and enhance the prognosis of individuals with cancer.

## AUTHOR CONTRIBUTIONS

L. X. W. and Z. C. Y. contributed to the conceptualization, data gathering, and writing of the manuscript. Z. Z. H. contributed to data gathering. X. P. and F. W. Y. contributed to the conceptualization, revising of the manuscript, and securing research funding. All authors read and approved the final paper.

## CONFLICT OF INTEREST STATEMENT

The authors declare that they have no conflict of interest.

### ETHICS STATEMENT

Not applicable.

## Data Availability

Not applicable.
